# A neo-W chromosome in a tropical butterfly links colour pattern, male-killing, and speciation

**DOI:** 10.1098/rspb.2016.0821

**Published:** 2016-07-27

**Authors:** David A. S. Smith, Ian J. Gordon, Walther Traut, Jeremy Herren, Steve Collins, Dino J. Martins, Kennedy Saitoti, Piera Ireri, Richard ffrench-Constant

**Affiliations:** 1 Natural History Museum, Eton College, Windsor SL4 6DW, UK; 2 BirdLife International, Africa Partnership Secretariat, Box 3502-00100, Nairobi, Kenya; 3 Department of Zoology, National Museums of Kenya, Box 4068-00100, Nairobi, Kenya; 4 Institut für Biologie, Zentrum für medizinische Struktur- und Zellbiologie, Universität Lübeck, Ratzeburger Allee 160, 23538 Lübeck, Germany; 5 Emerging Infectious Diseases Lab, ICIPE, Box 30772-00506, Nairobi, Kenya; 6 African Butterfly Research Institute (ABRI), Box 14308-0800, Nairobi, Kenya; 7 Insect Committee of Nature Kenya, Box 24467-00100, Nairobi, Kenya; 8 Department of Zoological Sciences, Kenyatta University, Box 43844-00100, Nairobi, Kenya; 9 Centre for Ecology and Conservation, University of Exeter, Penryn Campus, Penryn, Cornwall TR10 9EZ, UK

**Keywords:** *Danaus chrysippus*, male-killing, neo-W chromosome, colour pattern, speciation, *Spiroplasma*

## Abstract

Sexually antagonistic selection can drive both the evolution of sex chromosomes and speciation itself. The tropical butterfly the African Queen, *Danaus chrysippus*, shows two such sexually antagonistic phenotypes, the first being sex-linked colour pattern, the second, susceptibility to a male-killing, maternally inherited mollicute, *Spiroplasma ixodeti*, which causes approximately 100% mortality in male eggs and first instar larvae. Importantly, this mortality is not affected by the infection status of the male parent and the horizontal transmission of *Spiroplasma* is unknown. In East Africa, male-killing of the Queen is prevalent in a narrow hybrid zone centred on Nairobi. This hybrid zone separates otherwise allopatric subspecies with different colour patterns. Here we show that a neo-W chromosome, a fusion between the W (female) chromosome and an autosome that controls both colour pattern and male-killing, links the two phenotypes thereby driving speciation across the hybrid zone. Studies of the population genetics of the neo-W around Nairobi show that the interaction between colour pattern and male-killer susceptibility restricts gene flow between two subspecies of *D. chrysippus*. Our results demonstrate how a complex interplay between sex, colour pattern, male-killing, and a neo-W chromosome, has set up a genetic ‘sink' that keeps the two subspecies apart. The association between the neo-W and male-killing thus provides a ‘smoking gun' for an ongoing speciation process.

## 1. Introduction

Many arthropod species are infected with maternally inherited endosymbionts that induce a shift in the sex ratio of their hosts by killing males, so-called ‘male-killing’. Male-killing can increase infected female fitness at the expense of non-transmitting infected males by increasing the resources available to females, either by reducing inter-sibling competition or via cannibalism [1]. Endosymbionts that have near-perfect transmission may reduce the effective population size (*N*_e_) by a factor that approximates to the proportion of uninfected individuals in the population [2]. Theoretical studies of male-killing endosymbionts, where hybridizing subpopulations interbreed, have shown that local adaptation can be strongly impeded in the subpopulation with the more biased population sex ratio [3]. Further, it has been shown that both genetic drift and genetic influx are enhanced by male-killers; in cases where a nuclear gene is under selection (such as one which controls colour pattern), the two effects are unbalanced and the infection may have conspicuous effects [4].

One useful group of insects in which to examine the effects of male-killing are the butterflies. In East Africa, the African Queen butterfly, *Danaus chrysippus* (L.)—generation time one month, maximum adult lifespan around two months—is infected by a vertically transmitted, maternally inherited endosymbiotic bacterium, *Spiroplasma ixodetis*, that causes early mortality in males at the point of hatching from the egg, or occasionally in the first instar larva [5–10]. In the absence of suppression, vertical transmission of *Spiroplasma* in the Nairobi region of Kenya is 99.8% efficient (*n* = 1 240 offspring, *n* = 69 broods; electronic supplementary material, table S1). For *Spiroplasma* that are endosymbiotic, transmission is primarily vertical and there does not appear to be simultaneous reliance on horizontal and vertical transmission strategies. *Spiroplasma*-infected males which are assumed to carry a suppressor gene are normally rare and occur only seasonally. It is unknown if their sexual fitness is compromised.

**Figure 1.**
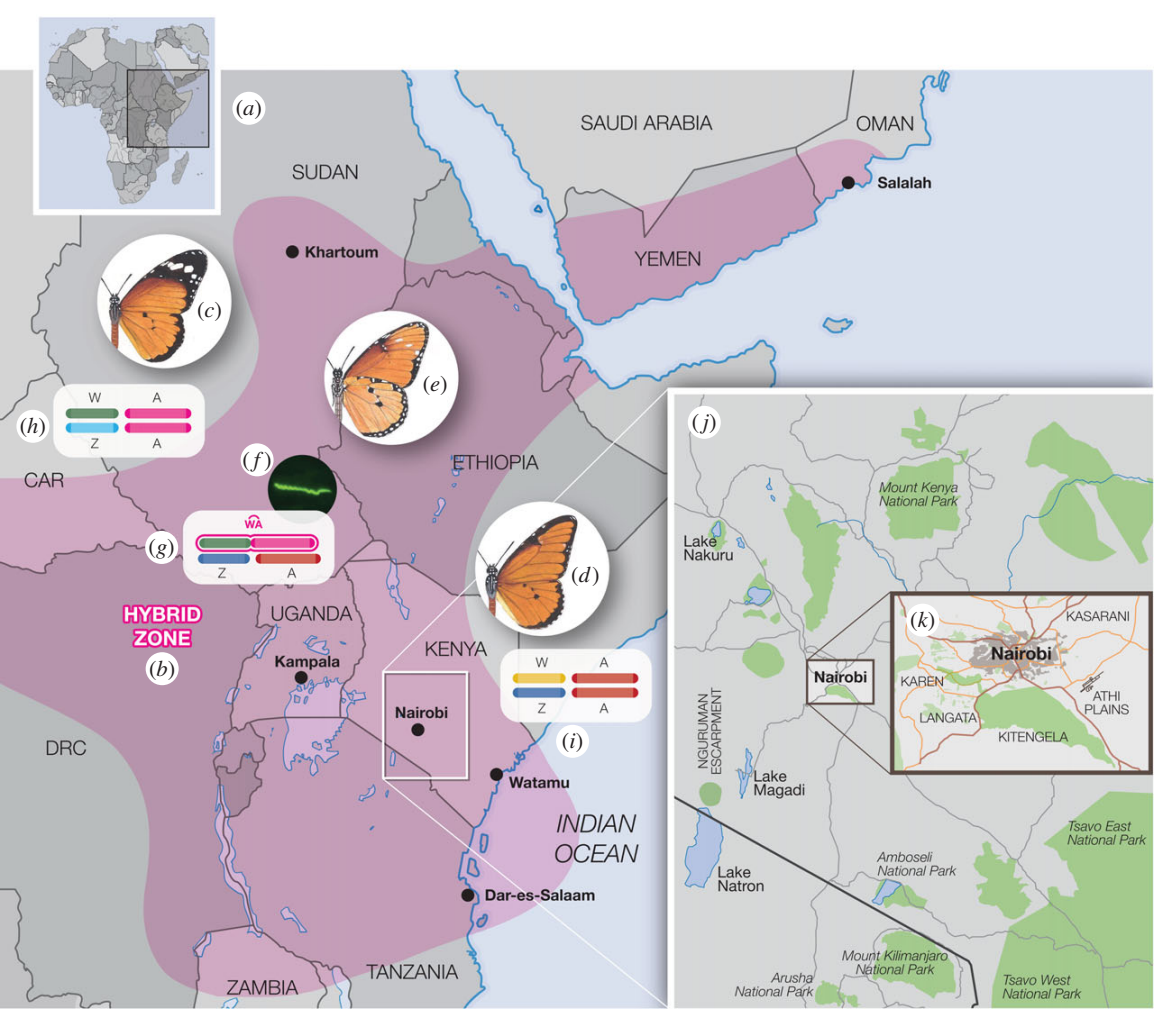
Map of the hybrid zone and the location of the different *Danaus* subspecies studied. (*a*) Map of East Africa showing the location of samples, the approximate position of the hybrid zone (*b*) and the distribution of *Danaus chrysippus* subspecies *chrysippus* (*c*) and *dorippus* (*d*). The hybrid form *D. c. transiens* (underside shown in *e*) is confined to the hybrid zone, as are the endosymbiont *Spiroplasma ixodetis* (*f*) and the ‘fused’ neo-W karyotype (*g*). Outside the hybrid zone the wild-type ‘unfused’ (Wu) karyotypes for *chrysippus* (*h*) and *dorippus* (*i*) are fixed. Sampling sites mentioned in the text are shown in the insets, southern Kenya (*j*) and the environs of Nairobi (*k*). The symbol A refers to the autosome carrying the *C* locus.

Alongside their differing susceptibility to male-killing, the African Queen also varies in colour pattern across its range. In fact, ever since Poulton [11] noted that two colour morphs could be bred from the same parents, *D. chrysippus* has been considered ‘polymorphic’. However, our long-term studies at three sites around Nairobi, Kenya (figure 1*a*,*j* and *k*), have established that in fact at this location the butterfly comprises two essentially parapatric (though migratory) subspecies. *D. c. chrysippus* and *D. c. dorippus* (here termed simply *chrysippus* and *dorippus*, figure 1*c*,*d*) each have an individual colour pattern controlled by a single autosomal locus *C* [12], *dorippus* being *CC* and *chrysippus cc*. The F_1_ hybrid *transiens* (*Cc*) closely resembles *dorippus* but is usually phenotypically detectable (figure 1*e*) [12]. The subspecies make frequent contact across East Africa creating colour polymorphisms within a mosaic of hybrid clines centred on Nairobi but reaching south to Tanzania [13], east into Uganda [5,8,10], and north to Sudan [10], Ethiopia [14], and Oman [15]. Outside the hybrid zone, both subspecies are monomorphic for the *C* locus through their separate ranges (figure 1, [16,17]). Previous investigations have explored relationships between colour pattern, male-killing, and sex-linkage. Throughout the hybrid zone female-biased sex ratios, driven by male-killing and marked by hybrid excess, predominate [16,17]. Two phenotypes are associated, the first being colour pattern, which may be sex-linked or independent of sex and the second is susceptibility to the male-killing *Spiroplasma*, or immunity to it [8–10].

We have previously found that the *c*-autosome of *chrysippus* females strictly segregates with the W chromosome within the hybrid zone and have, therefore, postulated that a W-autosomal fusion (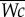) had occurred [18–20], physically linking the female determining W chromosome with the colour pattern locus *C*. Further, we have also found within the hybrid zone that *chrysippus* females and their *transiens* daughters are all infected with the male-killing *Spiroplasma* and thus produce all-female broods whereas, by contrast, *dorippus* females are largely uninfected by *Spiroplasma* and, therefore, continue to produce both males and females. The *D. chrysippus* hybrid zone is arguably an empirical example of the type of genetic ‘sink’ described in theoretical studies [3,4] which arises between hybridizing populations, one of which is infected and the other not. The aims of the present study were, therefore, to look at five specific factors likely to govern the population genetics of the hybrid zone. (i) To demonstrate, using cytogenetics, that an autosome has indeed become fused to the female-specific W-chromosome, as previously postulated. (ii) To document the frequency of *Spiroplasma* infection in the hybrid zone and to try to understand how this fusion maintains the highly female-biased sex ratios found. (iii) To investigate any possible relationship between population density and sex ratio. (iv) To document, via spermatophore count per female, potentially different rates of mating between hybrids and pure-bred individuals in the hybrid population. (v) Finally, to examine whether sexual selection or mate choice (assortative, random, or disassortative) within the hybrid zone affects sex ratio.

Here, in a cytogenetic analysis using fluorescent *in situ* hybridization (FISH) to visualize telomeres, we show that in *chrysippus* females the W chromosome is indeed fused to an autosome, forming a trivalent with the Z chromosome and the autosome in meiosis, whereas in *dorippus* females W/Z and autosomes pair independently, as does the Z and all the autosomes in all males. We also show that in a female-dominated hybrid zone site at Kitengela, immigrant *dorippus* males are relatively immune to male-killing and that nearly all females are inseminated despite outnumbering males by around 5 : 1. Paradoxically, the female-biased hybrid population has a *stable* sex ratio which withstands perturbations induced by weather, population density, or immigration and reinforces a barrier to gene flow between two nascent species that has endured for at least 40 years around Nairobi [15–17]. The extraordinary effectiveness of this barrier is demonstrated by field data showing a rapid return to the prevailing female-biased sex ratios after a seasonal influx of *Spiroplasma*-infected and presumably male-killer-resistant males.

## 2. Material and methods

### (a) Cytogenetics

Male metaphase I and II cells were obtained from testis cysts of pupae and last-instar larvae and female pachytene cells from the tips of ovarioles of adult females. Fixation in Carnoy's fluid (ethanol : chloroform : acetic acid, 6 : 3 : 1) was followed by spreading in 60% acetic acid and staining with 4′6-diamidino-2-phenylindole (DAPI). Female metaphase I was prepared from mature eggs by fixation in Carnoy's fluid, squashing in 60% acetic acid, freezing in liquid N_2_, flipping off the coverslip with a scalpel (dry ice method), post-fixation in ethanol, and staining with DAPI. Telomeres were visualized by FISH with a (TTAGG)n probe generated by substrate-free PCR (primers: TAGGTTAGGTTAGGTTAGGT and CTAACCTAACCTAACCTAAC) and labelled with Orange-dUTP.

### (b) *Spiroplasma* screening

DNA from adult *D. chrysippus* was extracted from a 2 mm^2^ piece of thorax tissue using the Puragene DNA purification kit (Quiagen, Valencia, CA, USA). DNA was extracted using three times the suggested volume for a single *Drosophila* and then hydrated in 70 µl ddH_2_O. Previously described *ixodetus*-clade specific primers (SpixoF and SpixoR) and PCR cycling conditions [21] were used to amplify an 810 bp region of *Spiroplasma* 16S rDNA to confirm the presence of *Spiroplasma*. As a positive control for DNA quality, a region of host mitochondrial DNA (mtDNA) was amplified using primers LCO1490 and HCO2198 [22].

### (c) Field sampling

The new field data from Kitengela comprise samples of flying adult butterflies collected by net in 19 2 h sessions from May 2013 to September 2015 (electronic supplementary material, table S2). As most butterflies seen were caught, the samples are ± random. All butterflies were date marked and released at the point of capture. Mated pairs were recorded; 260 wild females of random genotype were dissected to count spermatophores.

### (d) A model for the relationship between sex ratio and the frequency of male-killing

A model for the relationship between the sex ratio of the population and the frequency of the male-killer or ‘MK’ (electronic supplementary material, figure S1) is derived from the following premises: (i) the sons of MK (neo-W) females all die, (ii) non-MK (W_u_) females bear equal numbers of sons and daughters, (iii) MK and non-MK females produce equal numbers of surviving daughters, and (iv) the migration of females (though not of males) is effectively zero.

## 3. Results

### (a) Cytogenetics

**Figure 2.**
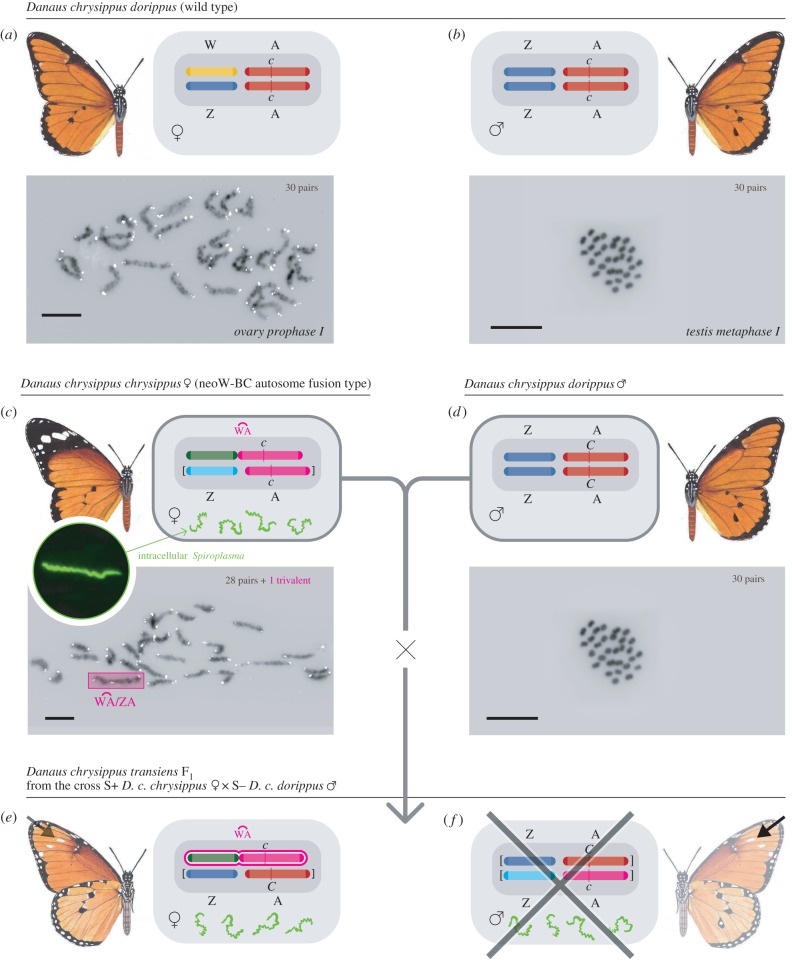
Colour phenotypes, genotypes, and karyotypes of subspecies *D. c. chrysippus* (*cc*), *D. c. dorippus* (*CC*), and the hybrid form *transiens* (*Cc*) in the Nairobi region. (*a–d*) Meiosis prophase I pachytene chromosome pairs from female ovaries (*a,c*) and metaphase I pairs from male testes (*b,d*): *Danaus chrysippus dorippus*, *a* female, *b* male, 30 bivalents (2*n* = 60) in both sexes; *D. c. chrysippus*, *c* female, 28 bivalents, 1 trivalent (2*n* = 59); *d* male, (2*n* = 60) as *b*; the white dots in the female micrographs *a* and *c* are telomere signals. Scale bars represent 10 µm. (*e*,*f*) F_1_ progeny, female (*e*) and male (*f*) from a cross between a *Spiroplasma*-positive *D. c. chrysippus* female (*c*) and a *Spiroplasma*-negative *D. c. dorippus* male (*d*). The provenance of chromosomes is shown as red, *dorippus C*-autosome; pink, *chrysippus c*-autosome; dark blue, *dorippus* Z chromosome; light blue, *chrysippus* Z chromosome; yellow, W chromosome unfused; green, W chromosome fused. Wild-type bivalents (in *a*,*b*,*d*) comprise, respectively, two sex chromosomes (Z/Z or Z/W) with two autosomes carrying the *C* locus, whereas the 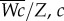 trivalent is found only in the mutant females *c* and *e*. Chromosomes in square brackets in females *c*, *e*, and male *f* are lost in successive generations of dead sons (marked with a cross in *f*). A few *f* males escape death by either immunity (MK suppression) or failed transmission of *Spiroplasma* [8]; most male survivors have the *transiens* (*Cc*) phenotype detectable by the white spots (arrowed in *e*,*f*) on the underside and/or scattered black scales on the upper side of the forewing apex.

Previous cross-breeding has suggested that the autosome carrying the *C* locus may have become linked to the female-specific W (and not the Z) chromosome via a previously undocumented fusion ([18–20]; electronic supplementary material, table S1). To test this hypothesis, we examined the cytogenetics of butterflies of known descent for colour pattern from within the Nairobi hybrid zone (figure 2). We found that the diploid chromosome complement in F_1_ *transiens* (*Cc*) females from Nairobi contains a fusion chromosome (a neo-W): 28 bivalents and a trivalent are present in female meiosis (2*n* = 59), whereas in meiosis of females from outside the hybrid zone 30 bivalents (2*n* = 60) are present (figure 2*b*,*d*; electronic supplementary material, table S3). This confirms our earlier prediction and explains the strict sex-linkage found only in the hybrid zone (figure 2*c*,*e*). As the neo-W pairs in meiosis I of oogenesis with Z and the *C*-autosome, the former becomes Z_1_ and the latter Z_2_ in a W/Z_1_Z_2_ trivalent. All males from several sources had 30 bivalents (2*n* = 60), in agreement with older data [23,24]. The dataset, therefore, directly supports our earlier hypothesis that an autosome carrying the *C* locus has indeed become directly fused to the female-specific W-chromosome.

### (b) *Spiroplasma* screening

In order to understand how infection with the male-killing *Spiroplasma* drives the female-biased sex ratios found within the hybrid zone, we typed 87 butterflies (72 females and 15 males) from the Kitengela field site for *Spiroplasma* (electronic supplementary material, table S4).

The high infection rate in female adults (87.9%, *n* = 72) at Kitengela, equally across all genotypes, is expected from the high frequency throughout the Nairobi region of the neo-W chromosome—78.7% of broods (*n* = 127) from wild females with sex-linkage for colour pattern and sex. The frequency of the male-killer is estimated from the proportion of wild females that produced all-female progenies in females as 71.7% (*n* = 127) ([18] and K Saitoti, IJ Gordon, and DAS Smith 2015, unpublished data) and is similar to that observed in 2009–2010 at Kasarani [20] (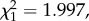 *p* = 0.158). However, Kitengela male samples had the highest ever recorded infection rates (66.7%) and significantly so (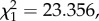 *p* < 0.0001) compared with the 2009–2010 Kasarani samples [20]. Comparing male infection rates for June (40.0%) and July (81.3%), the increase approaches significance (exact *p* (one-tailed) = 0.115). The increasing frequency of *Spiroplasma*-infected but male-killer-resistant males is reflected in a significant change in sex ratio from 86.6% female in May, through 82.1% in June, down to 72.4% in July (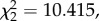 *p* = 0.005; electronic supplementary material, table S5). Each of these changes takes place within the period of a single generation, generation time being approximately one month. It should be noted that horizontal transmission of *Spiroplasma* has never been observed when vertical transmission has been demonstrated.

We paid particular attention to infection rates in the rarer males which appear after the rains (May–July 2015) and then appear to vanish over time. The high frequency of infected males (which can only exist if they are resistant) at this time was unusual. As invaders do not bring *Spiroplasma* from their population of origin, the infected males were probably first generation survivors of immigrants carrying suppressor genes. The fact that 85% of new males were of *CC* genotype, otherwise rare in the hybrid zone, supports this interpretation. However, factors other than migration may influence the frequency of male-killing. First, high population density may increase sibling competition, which is thought to be a main contributory factor promoting the spread of male-killers [25]. Although *D. chrysippus* females lay their eggs singly on the milkweed host plant, thus diminishing the risk from sibling competition, paradoxically they preferentially select isolated plants, which may then become overloaded at times of high population density [26]. Second, if *Spiroplasma* is to any extent horizontally transmitted, as may result from egg cannibalism [18], then high levels of infection may follow as a direct consequence of high population density [25]. As coincidental horizontal and vertical transmission of *Spiroplasma* has never been observed, however, we consider this alternative unlikely.

Prior to a brief period of high population density at Kitengela in May–July 2015, 90.8% (*n* = 76) of broods obtained from wild *Cc* males mated with *cc* females (electronic supplementary material, table S1) had produced all-female progeny. Two months after the period of high population density (in September) all males had vanished from the hybrid zone and the sample had returned to 100% female (electronic supplementary material, table S5). The all-female population was, therefore, restored and all males eliminated in as little as two butterfly generations. The summary statistics of the *D. chrysippus* populations which have been sampled for the physical presence of *Spiroplasma* and/or the male-killing phenotype, on the one hand, and hybridism on the other (electronic supplementary material, table S6) shows that the two phenomena are far from independent (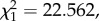 *p* < 0.0001). In conclusion, the high frequency of male-killing *Spiroplasma* and its association with the neo-W fusion within the hybrid zone rapidly cleanses the population of males. Following an unusual immigration of large numbers of males, they can be removed from the population within only a staggering *two* generations.

### (c) Field data

**Figure 3.**
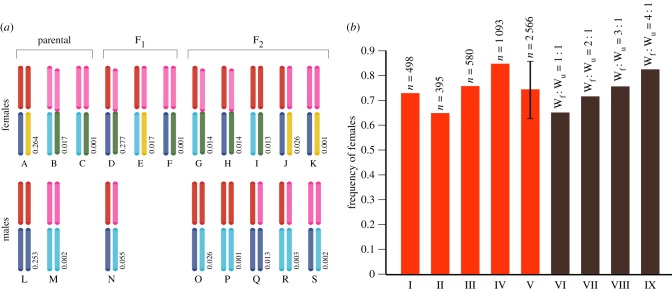
(*a*) The array of karyotypes in the F_2_ from founder crosses between *D. c. dorippus* and *D. c. chrysippus* populations in the Nairobi hybrid zone, displayed by sex and the generation in which they first appear. F_2_ karyotype frequencies are calculated on the following assumptions: (1) the sex ratio in the founder crosses is 1 : 1 in *dorippus* and 90% female in *chrysippus*, (2) the numbers of *dorippus* and *chrysippus* females in the founder population is equal, (3) all females are mated, (4) all male offspring of neo-W females die, and (5) there is neutrality and no migration. The upper pair of chromosomes comprises the C-autosomes, red for *dorippus* and pink for *chrysippus*. The lower pair shows the Z chromosomes of *dorippus* (dark blue) and *chrysippus* (light blue); the yellow W chromosome is always unfused (Wu) in *dorippus*, whereas in *chrysippus* the W chromosome shown in green may be unfused or fused to the C-autosome (neo-W).

We estimated the density of the Kitengela population on several occasions. Bailey Index estimates for population size [27] at the 5 hectare (ha) site varied between extremes of 27 ± 16.7 (5.4 per ha, 100.0% female) in October 2014 and 344 ± 229.5 (68.8 per ha, 86.6% female) in May 2015, an approximately 13-fold difference in density which has no apparent effect on the sex ratio (Exact *p* = 0.063). However, as the small number of recaptures produced high standard errors to the population measures, this conclusion is, therefore, tentative. Even so, the Kitengela sex ratio data (electronic supplementary material, table S2) conform to a historical (1986–2010) norm for the region (figure 3*b*,I–III). The mean sex ratio of 84.1% female (figure 3*b*,IV), though the highest recorded in the Nairobi region, nonetheless lies within the C_95_ limits for the regional mean (figure 3*b*,V).

### (d) Spermatophore counts

To test if females of different colour morphs experienced different rates of mating success, we counted the number of the male-transferred spermatophores found per female. Spermatophores per female at Kitengela (2013–2014, *n* = 260) averaged 1.73 ± 1.24 (range 0–6) with only 19 (7.3%) unmated; thus all females are expected eventually to mate. The sexual histories of *Cc* (*n* = 227) and *cc* females (*n* = 23) did not differ (*t*_258_ = 0.073, *p* > 0.99). The spermatophore count is significantly less than 3.50 ± 1.36 (*n* = 20) for a population in Ghana with a sex ratio of 1 : 1 (*t*_278_ = 6.126, *p* < 1.0 × 10^−4^). In conclusion, overall spermatophore counts per female do not differ between colour morphs within the hybrid zone.

### (e) Sexual selection

Finally, we used a period of unusually high male frequency to study the potential effects of sexual selection and to test if mating between colour morphs was assortative, random, or disassortative. In May–July 2015, the Kitengela population irrupted during heavy rains after a long period of drought. For the first time males were relatively numerous, and male–female courtship and successful mating was observed in the field. We seized the corresponding opportunity to study sexual selection (electronic supplementary material, table S7). The frequency of males caught *in copula* was 63.4% compared with 13.4% for females. In other words, males were nearly five times more likely to mate. This remarkable statistic attests to the unrelenting pressure to mate on males. Sexual selection for colour genotype was not significant for either sex (
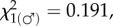
 *p* = 0.662; 
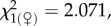
 *p* = 0.150). However, with numbers corrected for penetrance (electronic supplementary material, table S7), the majority of males *in copula* (63.3%) were *CC*, whereas among females 84.2% were *Cc*, 15.8% *cc*, and 0% *CC*. Thus, the absence of *CC* females enforces males to pair disassortatively for genotype. This result is counterintuitive for two reasons; first, in *Cc* and *cc* females, all of which are neo-W and male-killer-susceptible, the genes contributed by the male partner will all subsequently be eliminated [2–4]; second, the absence of *CC* females suggests that migration into the hybrid zone is substantially male-biased [3].

## 4. Discussion

This paper shows for the first time that a neo-W chromosome, a W-autosome ‘fusion’, is indeed responsible for the previously observed non-random segregation of colour pattern and sex in the African Queen butterfly and that the neo-W promotes the genetic separation of two incipient butterfly species across a hybrid zone. The data on morph frequencies and mated pairs also confirm for the first time that mating is disassortative within this hybrid zone despite being assortative outside it [7,28]. This paradoxical situation is entirely due to the restricted mate choice that results from male-killing and the neo-W fusion. Specifically, immigrant males are predominantly *CC* (*dorippus*), whereas all the infected females with which they mate are either *Cc* (*transiens*) or *cc* (*chrysippus*), and all their infected daughters carry the *c* allele. As mate choice in polymorphic populations is normally assortative for colour pattern [7,28], enforced disassortative mating potentially invokes sexual conflict [29–31]. Matrilineal descent of neo-W, combined with MK, would ensure that the progenies from such disparate pairings are overwhelmingly females of genotype 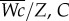 (electronic supplementary material, table S1) [17,18].

At this stage, we are uncertain as to exactly which autosome is fused to the female-specific W chromosome. However, what is clear is that this autosome carries loci (or indeed a single locus) controlling both colour pattern (the *C* locus) and susceptibility to the male-killing *Spiroplasma*. The high frequency of the neo-W fusion in the Kitengela population ensures that butterflies within the hybrid zone are predominantly female and that the sex ratio is stable despite seasonal influxes of male-killer-protected *Spiroplasma*-infected males. Hence, if these males carry a Z-linked suppressor gene, it would be lost in dead male offspring, thus perpetuating the female-biased sex ratio.

The African Queen has a generation time of approximately one month and a maximum adult lifespan of around two months. Remarkably, in this context, all females obtain at least one mating although their spermatophore count is significantly less than in a population with a 1 : 1 sex ratio. Thus, females in MK populations receive fewer (and possibly smaller) spermatophores from their exhausted male partners. As spermatophores contain nutrients and defensive pyrrolizidine alkaloids in addition to sperm [32], hybrid zone females are thereby disadvantaged. Moreover, as females outnumber males approximately 5 : 1, to achieve 1.7 pairings per female (as found at Kitengela) each male must mate an astonishing 8.5 times. Males will quickly be seduced by females, become entrapped and eventually exhausted [33]. Because within male-killer-susceptible females, all genes from the male partner are eliminated [2–4], strong pressure on the largely *CC* males to mate with unsusceptible *CC* females would be expected. However, as a result of male-biased migration the latter are absent. Indeed, if uninfected *CC* females were available this would quickly lead to restoration of a 1 : 1 sex ratio and this was never seen over 2 years of observation. Moreover, the highly unusual proportion of males caught *in copula* (63%, electronic supplementary material, table S7), all with male-killer-susceptible females, suggests that sexual selection is relaxed. As all males are in demand, all are expected to mate many times and their life expectancy must be curtailed. Furthermore, the paucity of males reduces the effective population size (*N*_e_) by more than or equal to 90% [2] and severe inbreeding is the likely outcome from an average of 4.3 females sharing each male partner. Therefore, the prognosis for the male-killer-susceptible females is to be both short-changed for nuptial gifts and inbred.

In figure 3, we show the karyotypes that result after a founding cross between ‘unfused’ *D. c. dorippus* males with 30 bivalents and *D. c. chrysippus* females with 28 bivalents and a W/Z_1_Z_2_ trivalent. The array assumes all possible crosses within the hybrid population after the first two rounds of mating. Most of the rare recombinants are heterozygotes for either *C*-autosomes, sex chromosomes or both, and arguably unfit. However, karyotype J with an unfused W chromosome is probably the standard karyotype in *chrysippus* populations outside the hybrid zone, which, in the absence of the neo-W and male-killing, should be subject to Fisherian selection for a balanced sex ratio [34]. This suggests that once free from the hybrid zone, *chrysippus* populations should quickly revert to 1 : 1 sex ratios.

The neo-W fusion is apparently able to avoid suppression and ensures that male immigration does not reduce the high frequencies of females, except in the very short term; in effect the neo-W fusion imposes a female-biased sex ratio on the Kitengela population. The influx of *dorippus* males in July 2015 reduced the sex ratio only for the duration of their lifespan (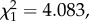 *p* = 0.043) and by September 2015, the female-biased sex ratio had been regained within just two generations (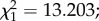 *p* = 0.0002; electronic supplementary material, table S5). The genetics of suppression are currently unclear, but, given the great selective advantage gained by male-killing suppressor genes [35], this extraordinary result is most easily explained if these genes are recessive and carried on the Z_2_ chromosome (approx. *C*-autosome) introduced by the immigrant males. This chromosome will be automatically eliminated in dead males within two generations, exactly as observed. Immigration by females from outside the hybrid zone does, however, have the potential to disrupt the sex ratio as it will always change the W_u_ : neo-W ratio (figure 3*b*). Ratios in the range 1 : 2 to 3 : 1 encompass a tipping point (electronic supplementary material, figure S1) between two adaptive peaks wherein the sex ratio switches rapidly from near normal to more than or equal to 80% female. When the neo-W is less common (less than or equal to 30%), the sex ratio remains relatively normal despite the presence of male-killing, and in practice (particularly in samples of active adults) will show no significant departures from 1 : 1. At Kitengela, the high frequency of the neo-W maintains sex ratios well above the tipping point and, even when males were most abundant in July 2015, adult female frequencies were never less than 70%. Moreover, all founding W_u_ : neo-W ratios in the range 1 : 1 to 1 : 4 fall inside C_95_ of the empirical mean for the Nairobi area (figure 3*b*,V), confirming the stability of the extreme sex ratios at Kitengela.

Within the hybrid mosaic the neo-W and male-killing are obligatorily linked as neither has ever been observed in the known absence of the other [18]. We speculate that the effect of the neo-W-male-killing package is to create a genetic ‘sink’ into which both *chrysippus* and *dorippus* male genomes are consigned, thus erasing their Z chromosomes and *C*-autosomes; these are precisely the expected locations of ‘speciation’ genes that would damage fitness in alien gene pools. The effectiveness of this genetic ‘sink’ is dramatically illustrated by the rapid return to all-female sex ratios observed at Kitengela in September after the incursion of male-killer suppressors in May–July 2015.

Our results are striking and unexpected for several reasons. First, they break Haldane's rule [36] which states: ‘when in the offspring of two animal races one sex is absent, rare, or sterile, that sex is the heterozygous [heterogametic] one’. Haldane's rule does not apply here because in *D. chrysippus* it is the *homogametic* males that die in the hybrid zone. Second, albeit with a different mechanism, the evolutionary outcome is similar to other animal hybrid zones between incipient species—stickleback fish [37], *Drosophila* or *Anopheles* [38], *Podisma* grasshoppers [39]—where gene flow is obstructed by hybrid male sterility, or in *Heliconius* butterflies by hybrid female sterility [40]. Third, unlike all the foregoing, the *D. chrysippus* genetic sink comprises a neo-W conserved by linkage in heterogametic females. It is, therefore, a reinforcement mechanism promoting speciation of a type that has not previously been described.

Finally, while we have yet to probe the molecular basis of this story, our results suggest two interesting possible alternatives for the future fate of the neo-W chromosome in the African Queen. The first alternative is suggested by the observation that *Eurema* butterflies carrying feminizing *Wolbachia* (*w*Fem) lack W chromatin and, therefore, appear to have ‘lost’ their W chromosome [41]. This supports an overarching hypothesis whereby infection with a microorganism (albeit *Wolbachia* or *Spiroplasma*) can render the W chromosome somehow redundant and hence either gradually lost (as in *Eurema*) or, in our case, translocated to an autosome to form a neo-W. Alternatively, and in the specific case of the African Queen, the physical act of fusing the W chromosome to an autosome may also disrupt an autosomal locus controlling both colour pattern and sex. To this end, we suggest a simple and testable hypothesis that both male-killer susceptibility and colour pattern are controlled by the gene *doublesex* [42] which is autosomal but has become subsequently female-linked by the formation of the neo-W of the African Queen. We note that *doublesex* has recently been shown to control not only colour polymorphism in another butterfly, *Papilio polytes* [43], but can also change the penetrance of male-killing via manipulation of alternative splicing within the gene itself [44]. If *doublesex* is indeed disrupted in the formation of the neo-W this would help explain why we see changes in both colour pattern and susceptibility to male-killing within the hybrid zone, as both may actually be controlled by the *same* locus. Taken together, our data support a hypothesis that two sexually antagonistic traits have been translocated to the newly formed female-specific neo-W chromosome of this butterfly and are now driving its speciation around Nairobi. However, any local advantage enjoyed by the neo-W may in reality be transitory and rapidly erode because Muller's ratchet will be expected to ensure the pervasive loss of autosomal genes and subsequent rapid degeneration of the neo-W chromosome [45–47].

## Supplementary Material

Smith et al ProcB Supplemental

## Data accessibility

The datasets supporting this article have been uploaded as part of the electronic supplementary material.

## References

[1] Hurst GDD, Majerus MEN 1993 Why do maternally inherited microorganisms kill males. Heredity 71, 81–95. (10.1038/hdy.1993.110)

[2] Engelstädter J 2010 The effective size of populations infected with cytoplasmic sex-ratio distorters. Genetics 186, 309–320. (10.1534/genetics.110.120014) 20592260 PMC2940295

[3] Kobayashi Y, Achaz G, Telschow A 2011 Effect of parasitic sex-ratio distorters on host gene frequencies in a mainland-island context. J. Evol. Biol. 24, 1695–1705. (10.1111/j.1420-9101.2011.02296.x) 21605214

[4] Telschow A, Engelstädter J, Yamamura N, Hammerstein P, Hurst GDD 2006 Asymmetric gene flow and constraints on adaptation caused by sex ratio distorters. J. Evol. Biol. 19, 869–878. (10.1111/j.1420-9101.2005.01049.x) 16674583

[5] Owen DF, Chanter DO 1968 Population of tropical African butterflies. 2. Sex ratio and polymorphism in *Danaus chrysippus* L. Rev. Zool. Bot. Afr. 78, 81–97.

[6] Smith DAS 1975 All-female broods in *Danaus chrysippus* L. and their ecological significance. Heredity 34, 363–371. (10.1038/hdy.1975.45) 1056323

[7] Gordon IJ 1984 Polymorphism of the tropical butterfly *Danaus chrysippus* L. in Africa. Heredity 53, 583–593. (10.1038/hdy.1984.116)

[8] Jiggins FM, Hurst GDD, Jiggins CD, Schulenburg JHGvD, Majerus MEN 2000 The butterfly *Danaus chrysippus* is infected by a male-killing bacterium. Parasitology 120, 439–446. (10.1017/S0031182099005867) 10840973

[9] Herren J, Gordon IJ, Holland PWH, Smith DAS 2007 The butterfly *Danaus chrysippus* (L.) in Kenya is variably infected with respect to genotype and body size by a maternally transmitted male-killing symbiont (*Spiroplasma*). Insect. Sci. Appl. 27, 62–69.

[10] Hassan SSM, Idris E, Majerus MEN 2012 Male-killer dynamics in *Danaus chrysippus* (L.) (Lepidoptera: Nymphalidae) in East Africa. Afr. J. Ecol. 50, 489–499 (10.1111/j.1365-2028.2012.01347.x).

[11] Poulton EB 1925 *Danaida chrysippus* L. and *D. dorippus* Klug, proved by breeding to be two forms of the same species. Proc. Entomol. Soc. Lond. 1924, cxix.

[12] Smith DAS 1975 Genetics of some polymorphic forms of the African butterfly *Danaus chrysippus* L. (Lepidoptera: Danaidae). Insect Syst. Evol. 6, 134–144. (10.1163/187631275X00235)

[13] Smith DAS 1998 Non-Mendelian segregation and variable penetrance of colour genes in the polymorphic butterfly *Danaus chrysippus* (L.). Heredity 80, 474–480. (10.1046/j.1365-2540.1998.00314.x)

[14] Cross P 2003 The butterflies of Wondo Genet: an introduction to the butterflies of Ethiopia. Addis Ababa, Ethiopia: Mega Printing Exercise.

[15] Lushai G, Allen JA, Goulson D, Maclean N, Smith DAS 2005 The butterfly *Danaus chrysippus* (L.) in East Africa comprises polyphyletic, sympatric lineages that are, despite behavioural isolation, driven to hybridization by female-biased sex ratios. Biol. J. Linn. Soc. 86, 117–131. (10.1111/j.1095-8312.2005.00526.x)

[16] Smith DAS, Owen DF, Gordon IJ, Lowis NK 1997 The butterfly *Danaus chrysippus* (L.) in East Africa: polymorphism, and morph-ratio clines within a complex, extensive and dynamic hybrid zone. Zool. J. Linn. Soc. 120, 51–78. (10.1111/j.1096-3642.1997.tb01272.x)

[17] Smith DAS, Gordon IJ, Depew LA, Owen DF 1998 Genetics of the butterfly *Danaus chrysippus* (L.) in a broad hybrid zone, with special reference to sex ratio, polymorphism and intragenomic conflict. Biol. J. Linn. Soc. 65, 1–40. (10.1111/j.1095-8312.1998.tb00349.x)

[18] Smith DAS 2014 African queens and their kin. Taunton, UK: Brambleby Books.

[19] Smith DAS, Gordon IJ, Allen JA 2010 Reinforcement in hybrids among once isolated semispecies of *Danaus chrysippus* (L.) and evidence for chromosome evolution. Ecol. Ent. 35, 77–89. (10.1111/j.1365-2311.2009.01143.x)

[20] Gordon IJ, Ireri P, Smith DAS 2014 Hologenomic speciation: synergy between a male-killing bacterium and sex-linkage creates a ‘magic trait’ in a butterfly hybrid zone. Biol. J. Linn. Soc. 110, 92–109. (10.1111/bij.12185)

[21] Duron O, Bouchon D, Boutin S, Bellamy L, Zhou L, Engelstädter J, Hurst GDD 2008 The diversity of reproductive parasites among arthropods: *Wolbachia* do not walk alone. BMC Biol. 6, 27 (10.1186/1741-7007-6-27) 18577218 PMC2492848

[22] Folmer O, Black M, Hoeh W, Lutz R, Vrijenhoek R 1994 DNA primers of mitochondrial cytochrome c oxidase subunit I from diverse invertebrates. Mol. Mar. Biol. Biotechnol. 3, 294–299. 7881515

[23] de Lesse H, Condamin M 1962 Formules chromosomiques de quelques Lépidoptères Rhopalocères du Sénégal. Bull. Inst. Fond. Afr. Noire 24, 464–473.

[24] Gupta Y 1964 Chromosome studies of some Indian Lepidoptera. Chromosoma 15, 540–561. (10.1007/BF00319989)

[25] Hurst GDD, Hurst LD, Majerus M 1997 Cytoplasmic sex ratio distorters. In Influential passengers (eds O'Neill SL, Hoffman A, Werren JH), pp. 125–154. Oxford, UK: Oxford University Press.

[26] Gordon IJ, Ireri P, Smith DAS 2014 Preference for isolated hosts facilitates invasion of *Danaus chrysippus* (Linnaeus, 1758) (Lepidoptera; Nymphalidae) by a bacterial male-killer *Spiroplasma*. Austral Entomol. 54, 210–216. (10.1111/aen.12113)

[27] Bailey NTJ 1951 On estimating the size of mobile populations from recapture data. Biometrica 38, 293–306. (10.1093/biomet/38.3-4.293)

[28] Smith DAS 1984 Mate selection in butterflies: competition, coyness, choice and chauvinism. In The biology of butterflies (eds Vane-Wright RI, Ackery PR), pp. 225–244. London, UK: Academic Press.

[29] Rice WR 1987 The accumulation of sexually antagonistic genes as a selective agent promoting the evolution of reduced combination between primitive sex chromosomes. Evolution 41, 911–914. (10.2307/2408899) 28564364

[30] Gavrilets S 2000 Rapid evolution of reproductive barriers driven by sexual conflict. Nature 403, 886–889. (10.1038/35002564) 10706284

[31] Perry JC, Rowe L 2014 The evolution of sexually antagonistic phenotypes. Cold Spring Harb. Perspect. Biol. 7, a017558 (10.1101/cshperspect.a017558) PMC4448611 26032715

[32] Dussourd DE, Harvis CA, Meinwald J, Eisner T 1989 Paternal allocation of sequestered plant pyrrolizidine alkaloid to eggs in the danaine butterfly *Danaus gilippus*. Cell. Mol. Life Sci. 45, 896–898. (10.1007/BF01954068) 2776861

[33] Charlat S, Reuter M, Dyson EA, Hornett EA, Duplouy A, Davies N, Roderick GK, Wedell N, Hurst GDD 2007 Male-killing bacteria trigger a cycle of increasing male fatigue and female promiscuity. Curr. Biol. 17, 273–277. (10.1016/j.cub.2006.11.068) 17276921

[34] Fisher RA 1930 The genetical theory of natural selection. Oxford, UK: Clarendon Press.

[35] Hornett EA, Moran B, Reynolds LA, Charlat S, Tazzyman S, Wedell N, Jiggins CD, Hurst GDD 2014 The evolution of sex ratio distorter suppression affects a 25cM genomic region in the butterfly *Hypolimnas bolina*. PLoS Genet. 10, e1004822 (10.1371/journal.pgen.1004822) 25474676 PMC4256269

[36] Haldane JBS 1922 Sex ratio and unisexual sterility in hybrid animals. J. Genet. 12, 101–109. (10.1007/BF02983075)

[37] Kitano J et al. 2009 A role for a neo-sex chromosome in stickleback speciation. Nature 461, 1079–1083. (10.1038/nature08441) 19783981 PMC2776091

[38] Michalak P 2013 Speciation: natural processes, genetics and biodiversity. New York, NY: Nova Biomedical.

[39] Veltsos P, Keller I, Nichols RA 2008 The inexorable spread of a newly arisen neo-Y chromosome. PLoS Genet. 4, e1000082 (10.137/journal.pgen.1000082) 18574519 PMC2435400

[40] Naisbit RE, Jiggins CD, Linares M, Salazar C, Mallet J 2002 Hybrid sterility, Haldane's rule and speciation in *Heliconius cydno* and *H. melpomene*. Genetics 161, 1517–1526. 12196397 10.1093/genetics/161.4.1517 PMC1462209

[41] Kern P, Cook JM, Kageyama D, Riegler M 2015 Double trouble: combined action of meiotic drive and *Wolbachia* feminization in *Eurema* butterflies. Biol. Lett. 11, 20150095 (10.1098/rsbl.2015.0095) 25948567 PMC4455736

[42] Kunte K, Zhang W, Tenger-Trolander A, Palmer DH, Martin A, Reed RD, Mullen SP, Kronforst MR 2014 *doublesex* is a mimicry supergene. Nature 507, 229–232. (10.1038/nature13112) 24598547

[43] Nishikawa H et al. 2015 A genetic mechanism for female-limited Batesian mimicry in a *Papilio* butterfly. Nat. Genet. 47, 404–409. (10.1038/ng.3241) 25751626

[44] Sugimoto TN, Ishikawa Y 2012 A male-killing *Wolbachia* carries a feminizing factor and is associated with degradation of the sex-determining system of its host. Biol. Lett. 8, 412–415. (10.1098/rsbl.2011.1114) 22219393 PMC3367752

[45] Charlesworth B, Charlesworth D 2000 The degeneration of Y chromosomes. Phil. Trans. R. Soc. Lond. B 355, 1563–1572. (10.1098/rstb.2000.0717) 11127901 PMC1692900

[46] Traut W, Sahara K, Marec F 2007 Sex chromosomes and sex determination in Lepidoptera. Sex. Dev. 1, 332–346. (10.1159/000111765) 18391545

[47] Sahara K, Yoshido A, Traut W 2012 Sex chromosome evolution in moths and butterflies. Chromosome Res. 20, 83–94. (10.1007/s10577-011-9262-z) 22187366

