# Supplementary material for: A neo-W chromosome in a tropical butterfly links colour pattern, male-killing, and speciation
Source: Proc Biol Sci. 2016 Jul 27;283(1835):20160821. doi: 10.1098/rspb.2016.0821 (PMC4971206; doi:10.1098/rspb.2016.0821)
Supplement: Smith et al ProcB Supplemental [file rspb20160821supp1.docx]

**Supplemental Information for *Smith et al*.**

Figure S1. The graph shows the frequency of females (*y*) in a hybrid population of *D. chrysippus* in the Nairobi district of Kenya as a function of the ratio W_u_: neo-W (*x*) in the founder females, where *y* = (*a* + *b*)/(2*a* + *b*), and *a* = *f*W_u_ (range 10-1), *b* = *f*neo-W (range 1-10). The model assumes neutrality and the absence of migration.

Table S1. Progenies of *D. chrysippus* obtained in the Nairobi region from females W-linked at the C locus [17-18]

__________________________________________________________________________________

MK *Cc* or *cc* ♀ × *Cc* ♂ (*n* = 69 broods) Non-MK *Cc* ♀ × *cc* ♂ (*n* = 7 broods)

| Offspring | Female | Male | Offspring | Female | Male |
| --- | --- | --- | --- | --- | --- |
| *CC* | 0 | 3 | *Cc* | 0 | 130 |
| *Cc* | 1237 | 0 | *Cc* | 119 | 0 |

MK = male-killing.

Notes. 1) The *c* allele invariably passes from mother to daughters. 2) The three male survivors in MK broods probably result from failed transmission of *Spiroplasma* [8]. 3) Male offspring which survive in the comparatively rare non-MK broods are assumed to carry a *Spiroplasma* suppressor gene.

Table S2. Observed frequencies of genotypes (within sex frequencies),

*corrected for penetrance*, in 19 field samples of adult *D. chrysippus* at

Kitengela, May 2013-September 2015

______________________________________________________________

Genotype frequencies

________________________________________________

*C- Cc cc n*

______________________________________________________________

Females 379 (41.2) 422 (45.9) 118 (12.9) 919

*0* (*174.5*) *801* (*452.0*) *118* (*292.5*) *919*

Males 148 (85.1) 19 (10.9) 7 (4.0) 174

*130* (*126.0*) *37* (*44.1*) *7* (*3.9*) *174*

________________________________________________­______________

Totals 527 (*300.5*) 441 (*496.1*) 125 (*296.4*) 1093

______________________________________________________________

Corrections for penetrance of *c* in *Cc* offspring are derived from cross-

breeding data in which 0.514 ± 0.015 (*n* = 1063) of heterozygotes are

visually identifiable.

Table S3. The cytogenetics of *D. chrysippus*. See also Figure 2.

| Source | Subspecies | Sex | Stage investigated^a^ | *n*^b^ | 2*n*^c^ | *N* |
| --- | --- | --- | --- | --- | --- | --- |
| Southeast Asia | *chrysippus* | ♂ | metaphase I + II | 30 | 60 | 2 |
| Israel | *chrysippus* | ♂ | metaphase I | 30 | 60 | 3 |
| South Africa | *orientis* | ♂ | metaphase I | 30 | 60 | 1 |
| Ghana | *alcippus* | ♂ | metaphase I + II | 30 | 60 | 1 |
| Watamu | *dorippus* | ♂ | metaphase I | 30 | 60 | 8 |
| Lake Turkana ♀  × Kakamega ♂ | NA | ♂ | metaphase I | 30 | 60 | 2 |
| Kitengela ♀  × Watamu ♂ | NA | ♂ | metaphase I | 30 | 60 | 1 |
| Watamu | *dorippus* | ♀ | pachytene + mitosis | 30 | 60 | 1 |
| Watamu | *dorippus* | ♀ | Pachytene | 30 | 60 | 4 |
| Watamu | *dorippus* | ♀ | Mitosis | 30 | 60 | 1 |
| Watamu | *transiens*^f^ | ♀ | metaphase I | 28 + 1^e^ | 59 | 1 |
| Watamu | *dorippus* | ♀ | pachytene +mitosis | 30 + B^d^ | 61 | 1 |
| Kitengela | *transiens*^f^ | ♀ | Pachytene | 28 + 1^e^ | 59 | 5 |
| Kitengela | *transiens*^f^ | ♀ | metaphase I | 28 + 1^e^ | 59 | 1 |
| Kitengela ♀  × Ghana ♂ | NA | ♀ | metaphase I | 28 + 1^e^ | 59 | 1 |

^a^meiosis unless stated otherwise; ^b^bivalents observed, or for mitotic data, inferred;

^c^diploid complement; ^d^B chromosome; ^e^trivalent; ^f^F_1_ from the cross *chrysippus* ♀

× *dorippus* ♂. Note. The difference between counts for wild type females from

Watamu (30 bivalents, *n* = 7) and mutant females from Kitengela (28 bivalents +

1 trivalent, *n* = 7) is highly significant, *P*_(one-tailed)_ = 1.2 × 10^–3^. With the exception

of females from Kitengela (*n* = 7) and Watamu (*n* = 2), all counts accord with

previous investigations from Sénégal [23] and India [24]. NA = not applicable.

Table S4. *Spiroplasma* (S) infection rates (% within-sex frequencies) in *D.*

*chrysippus* populations at two sites in the Nairobi region of Kenya.

_____________________________________________________________________

S+ S−

_________________ _________________

♂ ♀ *n* ♂ ♀ *n N*

_____________________________________________________________________

Kasarani 2009-10 [19] 3 90 93 41 25 66 159

(6.8) (78.3) (58.5) (93.2) (21.7) (41.5)

Kitengela 2015 14 58 72 7 8 15 87

(66.7) (87.9) (82.8) (33.3) (12.1) (17.2)

_____________________________________________________________________

Table S5. Sex ratios (% female) in the *D. chrysippus*

population at Kitengela, May-September 2015

____________________________________________

Males Females *n* % female

____________________________________________

May 17 110 127 86.6

June 32 147 179 82.1

July 51 134 185 72.4

September 0 40 40 100.0

____________________________________________

Totals 100 431 531 81.2

____________________________________________

Table S6. Summary of the association between

hybridism and *Spiroplasma* and/or male-killing (S/MK)

at 43 sites throughout the range of *D.chrysippus* [18]

______________________________________________

S−/MK− S+/MK+ *n*

______________________________________________

Hybrids + 2 28 30

Hybrids − 11 2* 13

______________________________________________

Totals 13 30 43

______________________________________________

*JH, new data: one site in Ghana, one in South Africa.

Table S7. Sexual selection in *D. chrysippus* at Kitengela, May-July 2015,

(*phenotypes corrected for penetrance as in Table S2*)

_____________________________________________________________

Genotypes *C-* *Cc* *cc* *n*

_____________________________________________________________

Males *in copula* 40 (*32*) 8 (*16*) 4 52

Males not *in copula* 24 (*19*) 5 (*10*) 1 30

_____________________________________________________________

Total males 64 (*51*) 13 (*26*) 5 82

_____________________________________________________________

Females *in copula* 16 (*0*) 23 (*39*) 13 52

Females not *in copula* 141 (*0*) 141 (*282*) 53 335

_____________________________________________________________

Total females 157 (*0*) 164 (*321*) 66 387

_____________________________________________________________

Totals 221 177 71 469

_____________________________________________________________
